# Chemical composition, antibacterial activity and related mechanism of valonia and shell from *Quercus variabilis* Blume (Fagaceae) against *Salmonella paratyphi* a and *Staphylococcus aureus*

**DOI:** 10.1186/s12906-019-2690-6

**Published:** 2019-10-18

**Authors:** Dan Zhou, Ze-Hua Liu, Dong-Mei Wang, Deng-Wu Li, Li-Na Yang, Wei Wang

**Affiliations:** College of Forestry, Northwest A & F University, Yangling, 712100 Shaanxi China

**Keywords:** *Quercus variabilis*, *S. paratyphi* a, *S. aureus*, Antibacterial mechanism

## Abstract

**Background:**

Plant secondary metabolites and phytochemicals that exhibit strong bioactivities have potential to be developed as safe and efficient natural antimicrobials against food contamination and addressing antimicrobial resistance caused by the overuse of chemical synthetic preservative. In this study, the chemical composition, antibacterial activities and related mechanism of the extracts of the valonia and the shell of *Quercus variabilis* Blume were studied to determine its potential as a safe and efficient natural antimicrobial.

**Methods:**

The phenolic compositions of valonia and shell extracts were determined by folin-ciocalteau colourimetric method, sodium borohydride/chloranil-based assay and the aluminium chloride method and then further identified by the reverse-phase HPLC analysis. The antibacterial activities of valonia and shell extracts were evaluated by the agar disk diffusion method and agar dilution method. The related antibacterial mechanism was explored successively by the membrane of pathogens effect, phosphorous metabolism, whole-cell proteins and the microbial morphology under scanning electron microscopy.

**Results:**

The n-butanol fraction and water fraction of valonia along with n-butanol fraction of the shell contains enrich phenolics including ellagic acid, theophylline, caffeic acid and tannin acid. The n-butanol fraction and ethanol crude extracts of valonia exhibited strong antibacterial activities against *Salmonella paratyphi* A (*S. paratyphi* A) and *Staphylococcus aureus* (*S. aureus*) with the DIZ values ranged from 10.89 ± 0.12 to 15.92 ± 0.44, which were greater than that of the *Punica granatum* (DIZ: 10.22 ± 0.18 and 10.30 ± 0.21). The MIC values of the n-butanol fraction and ethanol crude extracts of valonia against *S. paratyphi* A and *S. aureus* were 1.25 mg/ml and 0.625 mg/ml. The related antibacterial mechanism of n-butanol fraction and ethanol crude extracts of valonia may be attributed to their strong impact on membrane permeability and cellular metabolism. Those extracts exhibited strong antibacterial activity according to inhibit the synthesis of bacterial proteins and seriously change morphological structure of bacterial cells.

**Conclusions:**

The n-butanol fraction and ethanol crude extracts of valonia had reasonably good antibacterial activities against *S. paratyphi* A and *S. aureus*. This study suggests possible application of valonia and shell as natural antimicrobials or preservatives for food and medical application.

## Highlights

● Valonia and shell from *Quercus variabilis* possessed rich phenolics and tannins.

● Antibacterial activity of valonia and shell extracts were studied.

● VEE and VBF exhibited the strongest inhibition on *S. paratyphi* A *and S. aureus* and were selected to investigate the antibacterial mechanism.

● Integrity and permeability of cell membrane, phosphorus consumption, SDS-PAGE of protein patterns and cell microstructure were determined.

## Background

Plant secondary metabolites, or phytochemicals produced that exhibit antibacterial, antioxidant and antineoplastic activities have long been adopted to develop novel, safe and efficient natural antimicrobials for alternative medicine [1, 2]. Food contamination caused by the pathogenic bacteria such as *Escherichia coli, Listeria monocytogenes, Salmonella, Staphylococcus* affect food quality and represent a serious health risk to human intestines, urinary tract infection and food poisoning [3–6]. However, some chemical synthetic preservatives used in food control may cause respiratory diseases or other health risks [7, 8]. Overuse of antibiotics may result in the problem of antimicrobial resistance [9, 10]. Hence, recent research has focused on the search for natural antimicrobials from plants used as alternative medicines.

The Chinese cork oak, *Q. variabilis*, is a common warm-temperate deciduous broadleaf tree in East Asia and widely distributed in the north and east of China [11, 12]. *Q. variabilis* contributes a lot to the development of economy and ecosystem for its timber, cork and water conservation. As a significant energy tree species in China, the single-tree additive biomass models have been established to predict biomass or carbon stocks of cork oak forests in North China [13].

*Q. variabilis* cork is unique for its valuable properties including low density, high elasticity, good heat and acoustic insulation properties, which resistance to chemical and microbial attack for the production of stoppers for wine and thermal and/or acoustic insulation materials [14, 15]. The virgin and reproduction cork from *Q. variabilis* consist abundant suberin, lignin, extractives and polysaccharides [16, 17]. The acorn of *Q. variabilis* is rich in starch, soluble sugar, tannin, protein, grease and coarse fibre without tannins, which is usually used as feed for pigs, cows and goats [18, 19]. Leaves from different *Quercus* species contain polyphenols and natural triterpenoid that have the anti-inflammatory effects [20]. The acorn shell is used for produce activated carbon for the extraction as pigments. The valonia (cups of the acorn) contains approximately 28.45% tannin with a high purity of 77.64%. Ellagic acid and luteoic acid were isolated and identified from the tannin in valonia which is widely applied as medicine and food field [21–23]. Four species of lignins have been identified in the valonia and the physicochemical properties while the antioxidant activities have also been studied [24].

The phytochemistry of the valonia and shell from *Q. variabilis* have been studied however with limited research on their biological activities. Furthermore, due to the large annual production of *Q. variabilis*, the valonia and shell are a major source of wastage to their limited utilization. The objectives of this study were therefore to: (1) to determine the phytochemical composition, including the total content of phenolic, flavonoid and tannins of valonia and shell extracts from *Q. variabilis*; (2) measure the antibacterial activities of the extracts and (3) further investigation of the mechanisms of specific antimicrobial action of two effective extracts against selectively screened bacteria to support the development and utilization of these waste by-products.

## Methods

### Chemicals and microorganisms

Folin-Ciocalteu Reagent, chloramphenicol (Beijing Solarbio Co., Ltd., China); ellagic acid, galic acid monohydrate, caffeic acid, quercetin and theophyline (Shanghai Winherb Medical Science Co., Ltd., China); tannin acid (Tianjin Dongliqu Tianda Chemical Co., Ltd., China); ethanol, methanol, petroleum ether, ethyl acetate, n-butanol, dimethyl sulfoxide (DMSO) (Chengdu Kelong Chemical Co., Ltd., China); sodium carbonate (Na_2_CO_3_), sodium borohydride (NaBH_4_), aluminum chloride (AlCl_3_), hydrochloric acid, acetic acid, chloranil, vanillin, phosphomolybdic acid, sodium tungstate, and tetrahydrofuran (THF) (Tianjin Bodi Chemical Co., Ltd., China); chromatographic grade methanol and acetonitrile (Sigma-Aldrich Co., St. Louis, USA).

Muller Hilton Agar (MHA) and nutrient broth (NB) (Qingdao Hope Bio- Technology Co., Ltd., China).

*S. aureus*, *Listeria monocytogenes* (*L. monocytogenes*), *S. paratyphi* A, *Salmonella typhimurium* (*S. typhimurium*) and *Salmonella, L. enteritidis* (*S. enteritidis*) (Microbial Culture Collection Center of Guangdong Institute of Microbiology, China).

### Plant material and extracts preparation

The valonia and shell of *Q. variabilis* were collected from Northwest A&F University, Shaanxi province, China in September 2014. The voucher specimen was identified by the professor Dengwu Li and was deposited at Herbarium of the Northwest A&F University, Yangling, China (WUK 0489424). Plant materials were air-dried under shade at room temperature then stored at − 20 °C for further analysis. The valonia (1.0 kg) was extracted using 70% ethanol at room temperature for 12 h with a solid to liquid ratio of 1:10 and the extracted repeated 3 times. The ethanol crude extracts (VEE, 130.29 g) was filtered and evaporated to dryness and 6 g of VEE was stored for further analysis. The remaining VEE (124.29 g) suspended in water was further partitioned step by step by petroleum ether, ethyl acetate and n-butanol to give the petroleum ether extracts (VPEF, 2.65 g), ethyl acetate extracts (VEAF, 8.23 g), n-butanol extracts (VBF, 24.91 g) and water extracts (VWF, 66.53 g) [25].

Shell of *Q. variabilis* (500 g) was processed according to the above step and the ethanol crude extract (SEE, 6.00 g), petroleum ether extracts (SPEF, 0.52 g), ethyl acetate extracts (SEAF, 0.42 g), n-butanol extracts (SBF, 4.07 g) and water extracts (SWF, 3.97 g) were obtained.

### Phytochemical profiles of valonia and shell extracts

#### Determination of total phenolic (TPC) and flavonoid content (TFC)

The TPC was determined using the Folin-Ciocalteau colourimetric method [26]. 200 μL extracts (0.4 mg/ml) or different concentrations of gallic acid solution were mixed with 800 μL deionized water to which 200 μL Folin-Ciocalteu Reagent was added and boiled for 6 min. 2 ml of 7% Na_2_CO_3_ and 1.6 ml deionized water were added and left standing for 90 min at room temperature after which absorbance measured at 517 nm. TPC was expressed as mg gallic acid equivalent per 1 g (dry weight). All samples were analyzed in triplicate.

The TFC was determined by the sodium borohydride/chloranil-based assay in samples at the concentration of 2.0 mg/ml [26]. TFC was expressed as millimole quercetin equivalent per 100 g (dry weight). All the samples were analyzed in triplicate.

#### Determination of total tannin content (TTC)

The TTC was assessed using the aluminium chloride method [27]. 1.0 ml F-D chromogenic agent and 1.0 ml extracts (1.0 mg/ml) or different concentrations of tannin acid solution were mixed, 1.0 ml 1 mol/L Na_2_CO_3_ was then added to this and made up to volume using 80% ethanol. The solution was allowed stand for 30 min and the absorbance measured at 720 nm. TTC was expressed as mg tannic acid equivalent per 1 g (dry weight). All the samples were analyzed in triplicate.

#### Reverse-phase HPLC analysis of the main compounds

Samples were filtered through 0.22 μm membrane filters and then analyzed by using an Agilent Technologies 1260 series liqudi chromatograph (RP-HPLC). The quantification was carried out on a SB-C18 reverse phase column (5 μm, 4.6 by 250 mm) at ambient temperature. The mobile phases consisted of water (0.2% acetic acid, solvent A) and acetonitrile (solvent B). The gradient elution program was as follows: 5% B (0 min), 10% B (0–20 min), 20% B (20–30 min), 25% B (30–35 min), 40% B (35–45 min), 80% B (45–60 min). The mobile phase flow rate was kept at 0.8 ml/min. The injection volume was 20 μL and the chromatogram was measured at 254 nm. Analyses were performed in triplicate.

### Antibacterial activities of valonia and shell extracts

#### Agar disk diffusion method

The agar disk diffusion method was employed for the evaluation of antibacterial activities [28]. The extracts (5 mg/ml) were dissolved in 2% DMSO. The ethonal extracts of *Punica granatum* and ellagic acid were used as positive controls and the DMSO as negative control. The zone of the inhibition (DIZ) was measured in millimeters and ranked as follows: not sensitive for zone diameters equal to 8 mm or below; sensitive for zone diameters between 8 and 14 mm, very sensitive for zone diameters between 14 and 20 mm. Reported inhibition zones were the average values calculated triplicate.

Minimum inhibition concentration (MIC) and minimum bactericidal concentration (MBC).

The MIC of ten polarity fractions of valonia and shell from *Q. variabilis* were determined by agar dilution method [29]. A series of two-fold dilutions of each extracts was dissolved in 2% DMSO to obtain the concentration from 1.56 to 100 mg/ml. Each of the tested sterile petri dishes contained 18 ml MHA and 2 ml extracts. 3 μL bacterial inoculum (10^6^ CFU/ml) was placed on plate for each dilution and then incubated at 37 °C for 24 h. Negative control was performed using 2% DMSO. The MIC was the lowest concentration of the extracts in which no growth on the plate was observed. Then the plates beyond MIC value continued incubated at 37 °C for 24 h. The concentration at which no visible growth was seen was determined as the MBC [30].

Two effective antibacterial extracts were selected to investigate the antibacterial mechanism against two screened bacteria.

### The effect of VBF and VEE on the membrane of pathogens

#### Leakage of proteins

The bacterial suspension (10^8^ CFU/ml) was added into the NB with 1 × MIC VEE and VBF. The control was conducted with 2% DMSO. The cultures were incubated at 37 °C with shaking at 180 rpm for 1, 4, 8 and 24 h. Then centrifuged at 10,000×g for 15 min and the precipitation was added 500 μL NaCl (0.9%) and then centrifuged at 10,000×g for 5 min. The supernatant liquid was immediately determined by micro protein assay [31].

#### Leakage of nucleic acid

The bacterial suspension (10^8^ CFU/ml) was added into the NB at 37 °C with shaking at 180 rpm overnight. Then centrifuged at 10,000 g for 5 min and the precipitation were diluted with 7 mL NaCl (0.9%). The mixture was exposure to 1 × MIC VEE and VBF for 1, 4, 8 and 24 h. The cultures were immediately filtered with 0.2 μm syringe filters to remove the bacteria. Then the supernatant was diluted appropriately and optical density at 260 nm was recorded [32].

#### Leakage of total sugar in the medium

The bacterial suspension (10^8^ CFU/ml) were added into the NB at 37 °C with shaking at 180 r/min overnight. 1 mL suspension was added into 7 ml NB with 1 × MIC VEE and VBF. The mixture was incubated for 1, 4, 8 and 24 h. The samples were centrifuged at 10,000 g for 5 min to obtain the precipitation. 50 μL of the precipitation and 200 μL anthrone were added and then cooled in ice water for 5 min. Then the mixture was water bathed for 10 min. The absorbance was measured at 620 nm by microplate reader [33].

### The effect of VEE and VBF on the phosphorous metabolism of bacteria

The bacterial suspension (10^8^ CFU/ml) was added into the NB at 37 °C with shaking at 180 rpm overnight. Then centrifuged at 5000 g for 10 min and the precipitation were diluted with 0.1 mol/L phosphate buffer (10^6^ CFU/ml). Add 2 ml glucose and 800 μL phosphoric acid standard solution into 2 ml bacterial suspension. Then 800 μL of VEE and VBF (1 × MIC) were added and the mixture were incubated for 0, 1, 2, 4, 6, 8 and 12 h. 0.1 ml suspension and 1 ml trichloroacetic acid-ferrous sulfate were mixed and stand for 10 min, then centrifuged at 5000 g for 5 min. 2 ml supernatant and 50 μL ammonium molybdate were mixed and placed in 30 °C for 15 min. The mixture was measured at 630 nm by microplate reader [34].

### SDS-PAGE of whole-cell proteins

SDS-PAGE of the bacterial proteins was carried out according to the method [35]. After exposure to 1 × MIC VEE and VBF for 24 h, the bacterial cells samples were centrifuged at 10,000 g for 5 min. The cell pellet was rinsed and washed three times with 0.01 mol/L phosphate buffer (PBS) and then collected by centrifugation at 5000 g for 5 min. The buffer was added into the cells mixture and boiled for 5 min and then cooled on ice. The control was run without the extracts. Then the supernatant of each sample was collected for the SDS-PAGE analysis. After electrophoresis, the gel was stained with coomassie brilliant blue R-250 and then decolorized to obtain the separated protein bands.

### Scanning electron microscopy (SEM) analysis

To determine the morphological changes of bacteria treated by the extracts, SEM studies were carried out [36]. Logarithmic growth phase cells of two tested bacteria were treated with 1 × MIC VEE and VBF. The control (without the samples) and samples were incubated at 37 °C for 24 h. The cells were harvested by centrifugation (10,000 g, 10 min) and washed twice with 0.1 M PBS and then fixed with 2.5% (v/v) glutaraldehyde in PBS overnight at 4 °C. After centrifugation, the cells were further dehydrated using a graded series of ethanol (30, 50, 70, 80, 90 and 100%) and drying with hexamethyl-disilazane. The samples were fixed on SEM support and then sputter-coated with gold under vacuum, followed by microscopic examinations using SEM.

## Results

### Contents of TPC, TTC and TFC

Plant polyphenols are considered to possess biological activities such as antioxidant properties and antibacterial activities. Results regarding TPC, TFC and TTC of valonia extracts (VEE, VPEF, VEAF, VBF and VWF) and shell extracts (SEE, SPEF, SEAF, SBF and SWF) from *Q. variabilis* are shown in Table 1. The contents of TPC were ranged from 68.64 to 711.46 mg GAE/g. The highest level of TPC was found in VWF (711.46 ± 7.32 mg GAE/g), followed by VBF (673.66 ± 1.22 mg GAE/g) and VEE (641.95 ± 4.88 mg GAE/g). The TPC of the rest of extracts phase were below 600 mg GAE/g. The TPC contents in valonia extracts were higher than that of shell, the TPC contents in VEE were higher than that of SEE as well as the VEAF and SEAF.

**Table 1:** Total phenolic, flavonoid and tannins content of valonia and shell from *Q. variabilis*

Organ	Extracts	TPC (mg GAE/g)	TFC (mmol QUE/100 g)	TTC (mg TAE/g)
Valonia	VEE	641.95 ± 4.88	19.77 ± 0.07	142.33 ± 0.51
	VPEF	188.05 ± 1.52	29.21 ± 0.02	40.56 ± 0.33
	VEAF	529.76 ± 2.44	27.37 ± 0.06	237.49 ± 1.84
	VBF	673.66 ± 1.22	16.98 ± 0.03	258.02 ± 3.34
	VWF	711.46 ± 7.32	22.01 ± 0.00	228.47 ± 1.50
Shell	SEE	461.49 ± 6.10	31.05 ± 0.01	138.10 ± 0.33
	SPEF	68.64 ± 3.68	24.63 ± 0.06	57.26 ± 1.00
	SEAF	426.71 ± 1.83	31.35 ± 0.05	205.25 ± 3.67
	SBF	593.17 ± 3.66	45.82 ± 0.02	253.35 ± 4.51
	SWF	512.68 ± 4.88	23.75 ± 0.03	218.11 ± 1.17

Each value represented in tables are means ± SD (*n* = 3).

The contents of TFC ranged from 16.98 to 45.82 mmol QUE/100 g. The highest level of TFC was found in SBF (45.82 ± 0.02 mmol QUE/100 g), followed by SEAF (31.35 ± 0.05 mmol QUE/100 g) and SEE (31.05 ± 0.01 mmol QUE/100 g). The TFC of the rest of extractions were below 30 mmol QUE/100 g. The TFC of the same solvent extract phase of shell were higher than that of valonia except petroleum ether extract.

The contents of TTC were ranged from 40.56 to 258.02 mg TAE/g and the highest level of it was found in VBF (258.02 ± 3.34 mg TAE/g), followed by SBF (253.35 ± 4.51 mg TAE/g), VEAF (237.49 ± 1.84 mg TAE/g). The TTC of the other extracts were below 230 mg TAE/g. The valonia extracts from same solvent possessed higher TTC contents than that of shell except of petroleum ether extract. It suggested that there were differences of chemical composition content among different parts of the same plant.

### Contents of four compounds by HPLC analysis

**Fig. 1:**
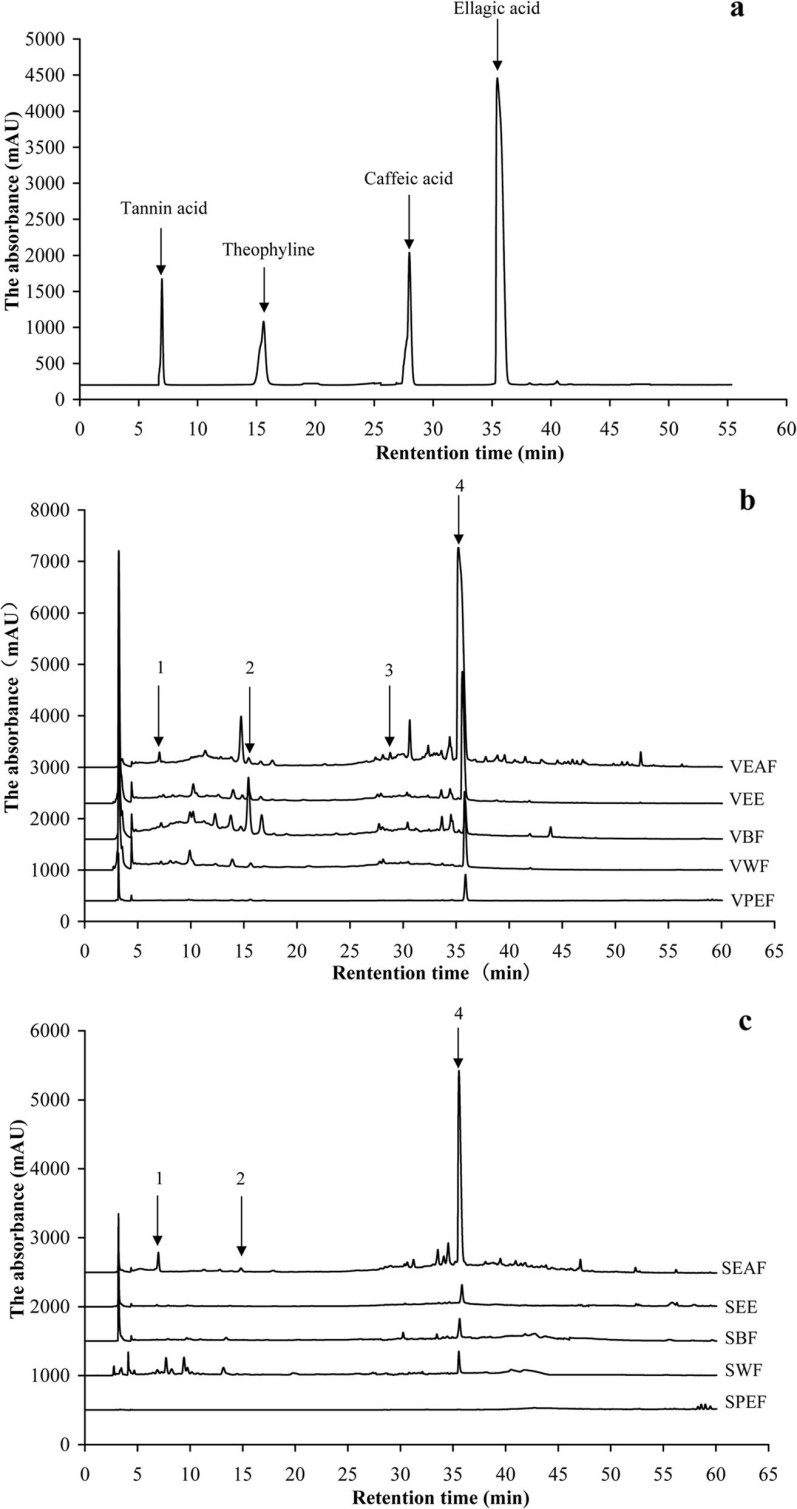
Analytical HPLC chromatogram of reference substances (**a**) and compounds of extracts of valonia (**b**) and shell (**c**) from *Q. variabilis*

Chromatograms of valonia and shell extracts from *Q. variabilis* were analyzed by HPLC method (Table 2). Four compounds in valonia extracts were identified as ellagic acid, tannin acid, theophylline and caffeic acid and the contents were determined (Fig. 1, Table 3). The precision of the analytical method was determined by assaying six replicates of the standard compounds, and the relative standard deviations (RSD) of the peak areas were estimated to be 0.20–0.41% (*n* = 6). The repeatability of the method was determined by injecting the same sample six times. The areas of the peaks were recorded, and the RSD of the areas varied from 1.11 to 2.75% (*n* = 6). To confirm the accuracy of the method, a recovery experiment was performed by mixing quantified samples with specific quantities of standard compounds. The average percentages of recovery of the four compounds ranged from 0.34 to 2.75% (*n* = 6).

**Table 2:** Method validation for the quantitative determination of four compounds using RP-HPLC

Peak no.	Compounds	Retention time	Regression equation	Precision experiment		Repeatability		Recovery experiment	
				Area of peak	RSD (%)	Area of peak	RSD (%)	Average recovery rate	RSD (%)
1	tannin acid	6.794	Y = 19,106x + 31.263 (R^2^ = 0.9996)	2492.6 ± 10.1	0.41	159.4 ± 33.44	2.09	105.57 ± 1.75	1.36
2	theophyline	15.605	Y = 30,252x-13.334 (R^2^ = 0.9995)	3753 ± 5.9	0.20	1346.8 ± 53.74	2.22	102.86 ± 1.25	1.18
3	caffeic acid	27.98	Y = 31,908x + 31.065 (R^2^ = 0.9994)	4172.2 ± 8.2	0.20	625.26 ± 17.25	2.75	98.36 ± 0.36	0.34
4	ellagic acid	35.447	Y = 206,229x + 1865.8 (R^2^ = 0.9984)	22,707.5 ± 103.3	0.34	20,841.7 ± 232.01	1.11	102.54 ± 2.83	2.75

Values were expressed in mean ± SD (*n* = 6).

**Table 3:** Contents of four compounds of valonia and shell extracts from *Q. variabilis*

Organ	Extracts	Compound (mg/g)			
		Ellagic acid	Tannic acid	Theophyline	Caffeic acid
Valonia	VEE	18.95 ± 0.20	2.21 ± 0.33	9.58 ± 0.27	2.64 ± 0.14
	VPEF	2.17 ± 0.04	0.14 ± 0.02	1.39 ± 0.03	0.05 ± 0.02
	VEAF	74.57 ± 3.02	13.41 ± 0.47	20.17 ± 0.003	1.74 ± 0.002
	VBF	3.08 ± 0.07	6.41 ± 0.76	66.23 ± 2.21	5.63 ± 0.57
	VWF	8.68 ± 0.25	2.72 ± 0.20	5.18 ± 0.33	3.23 ± 0.11
Shell	SEE	0.90 ± 0.11	1.09 ± 0.05	0.15 ± 0.005	0.36 ± 0.005
	SPEF	–	–	–	–
	SEAF	22.66 ± 0.23	16.96 ± 1.87	0.28 ± 0.002	0.33 ± 0.05
	SBF	1.02 ± 0.09	–	–	–
	SWF	0.55 ± 0.08	3.37 ± 0.06	1.36 ± 0.16	–

Each value represented in tables are means ± SD (*n* = 3).

In valonia and shell extracts, no targeted compound were detected in SPEF, while only ellagic acid (1.02 ± 0.09 mg/g) was tested in SBF. After extraction separation, VEAF exhibited the highest contents of ellagic acid (74.57 ± 3.02 mg/g), followed by SEAF (22.66 ± 0.23 mg/g) and VEE (18.95 ± 0.20 mg/g). Tannin acid was primarily concentrated in SEAF (16.96 ± 1.87 mg/g) and VEAF (13.41 ± 0.47 mg/g). Theophylline and caffeic acid were especially high in VBF, with the content of 66.23 ± 2.21 mg/g and 5.63 ± 0.57 mg/g. The valonia contained higher total content of compounds compared with the shell. Ellagic acid, theophylline and caffeic acid in valonia were higher that of shell from same solvent extraction.

### Antibacterial activities of valonia and shell extracts

#### Preliminary screening of strains

Natural phenolic products may exhibit a wide range of biological effects [8]. The results indicated that total phenolic and tannin were rich in the extracts of valonia and shell from *Q. variabilis.* Thus the antibacterial activities of the ethanol extracts against five bacteria were shown in Table 4. The diameters of inhibition zones (DIZ) were exerted by the various extracts towards challenged microorganisms. The control (2% DMSO) did not inhibit any of microorganisms tested. The results indicated that valonia was active against *S. paratyphi* A, with the DIZ of 12.37 mm, followed by *S. aureus* (10.89), *S. typhimurium* (10.32), *S. enteritidis* (9.52) and *L. monocytogenes* (9.36). In addition, *Punica granatum* peel was used as positive control due to its rich content of phenolics and flavonoids that displayed strong inhibition effects on the food-borne pathogens, including *L. monocytogenes*, *S. aureus* and *S. enteritidis* [37]. The ethanol extract of *Punica granatum* peel possessed strong activities against *L. monocytogenes* (DIZ 10.83 mm) than valonia and weaker antibacterial activities against the other four bacteria than valonia. The inhibition effects of shell extracts on *S. aureus* and *S. paratyphi* A were weaker than that of valonia. Chloramphenicol (5 μg/ml) as positive control was active against tested microorganisms, and the inhibition effects were 2–4 times to valonia and shell.

**Table 4:** Antibacterial activities of ethanol extracts of valonia and shell from *Q. variabilis*

Microorganisms	Inhibition zone (mm)				
	Valonia	Shell	*Punica granatum*	Chloramphenicol	DMSO
*S. aureus*	10.89 ± 0.12^a^	8.99 ± 0.22^a^	10.22 ± 0.18^a^	24.83 ± 0.61^a^	–
*S. paratyphi A*	12.37 ± 0.36^a^	9.62 ± 0.16^a^	10.30 ± 0.21^a^	29.96 ± 0.01^a^	–
*S. typhimurium*	10.32 ± 0.33^a^	8.69 ± 0.12^a^	9.95 ± 0.19^a^	27.10 ± 0.74^a^	–
*S. enteritidis*	9.52 ± 0.28^a^	8.56 ± 0.07^a^	9.35 ± 0.25^a^	33.96 ± 0.62^a^	–
*L. monocytogenes*	9.36 ± 0.32^a^	8.88 ± 0.08^a^	10.83 ± 0.17^a^	26.36 ± 0.85^a^	–

Each value represented in tables are means ± SD (*n* = 3). ^a^correlation is significant at the 0.01 level compared with the negative control (DMSO).

In conclusion, the results indicated that the extracts of valonia and shell from *Q. variabilis* were bioactive against *S. aureus* and *S. paratyphi* A. Thus the *S. aureus* and *S. paratyphi* A were chosen for further research of the antibacterial activities and related mechanism.

#### Antibacterial activities of ten extracts against *S. aureus* and *S. paratyphi* a

As shown in Table 5, the antibacterial activities of valonia and shell were measured. The DIZ against *S. aureus* were ranked as followed: VBF (12.26 mm) > VEAF (11.64 mm) > VEE (10.89 mm) > VWF (9.71 mm) > SEE (8.99 mm) > SWF (8.01 mm) > SEAF (7.97 mm) > SBF (7.06 mm). For *S. aureus*, the petroleum ether extract had no inhibition effects. SEAF and SBF was not sensitive to *S. aureus* for DIZ equal to 8 mm or below. The others expressed sensitive activity for DIZ between 8 and 14 mm. The antibacterial activities of valonia and shell against *S. paratyphi* A was ranked as followed: VBF (15.92 mm) > VEE (12.37 mm) > VEAF (12.26 mm) > VWF (10.25 mm) > SEE (9.62 mm) > SEAF (8.72 mm) > SWF (8.07 mm) > SBF (7.86 mm) > VPEF (7.62 mm). VBF possessed very sensitive activity to *S. paratyphi* A for DIZ between 14 and 20 mm. The antibacterial activity of VPEF and SBF against *S. paratyphi* A was not sensitive.

**Table 5:** Antibacterial activities of valonia and shell extracts from *Q. variabilis* against *S. aureus* and *S. paratyphi* A

Samples		Inhibition zone (mm)		MIC (mg/ml)		MBC (mg/ml)	
		*S. aureus*	*S. paratyphi* A	*S. aureus*	*S. paratyphi* A	*S. aureus*	*S. paratyphi* A
Valonia	VEE	10.89 ± 0.12^a^	12.37 ± 0.36^a^	0.625	1.25	0.625	1.25
	VPEF	ND	7.62 ± 0.43^a^	> 10	> 10	> 10	> 10
	VEAF	11.64 ± 0.43^a^	12.26 ± 0.12^a^	1.25	2.5	1.25	2.5
	VBF	12.26 ± 0.14^a^	15.92 ± 0.44^a^	0.625	1.25	0.625	1.25
	VWF	9.71 ± 0.18^a^	10.25 ± 0.14^a^	0.625	1.25	0.625	1.25
Shell	SEE	8.99 ± 0.22^a^	9.62 ± 0.16^a^	2.5	5	2.5	5
	SPEF	ND	ND	> 10	> 10	> 10	> 10
	SEAF	7.97 ± 0.11^a^	8.72 ± 0.14^a^	5	2.5	5	5
	SBF	7.06 ± 0.01^a^	7.86 ± 0.11^a^	2.5	2.5	2.5	2.5
	SWF	8.01 ± 0.05^a^	8.07 ± 0.09^a^	2.5	2.5	2.5	2.5
*Punica granatum*		10.22 ± 0.18^a^	10.30 ± 0.21^a^	–	–	–	–
Ellagic acid		10.24 ± 0.14^a^	11.45 ± 0.25^a^	–	–	–	–
DMSO		–	–	–	–	–	–

Each value represented in tables are means ± SD (*n* = 3). ^a^correlation is significant at the 0.01 level compared with the negative control (DMSO). *ND* Not determined with this extract.

The antibacterial properties of the extracts were also evaluated by the MIC and MBC values. The control (2% DMSO) did not inhibit any of microorganisms. Among the extracts, VEE, VBF and VWF had better antibacterial effect on *S. aureus* and *S. paratyphi* A with the MIC values of 0.625 mg/ml and 1.25 mg/ml. The MBC values were the same as MIC. Poor inhibitory activities were detected against the two strains in VPEF and SPEF (≥10 mg/ml). The antibacterial activities of valonia were stronger than shell extracts. The antibacterial activities showed that VEE and VBF had better inhibition effects on *S. aureus* and *S. paratyphi* A, so VEE and VBF were chosen to further study the mechanism.

### The effect of VBF and VEE on the membrane of *S. aureus* and *S. paratyphi* a

#### Leakage of proteins

**Fig. 2:**
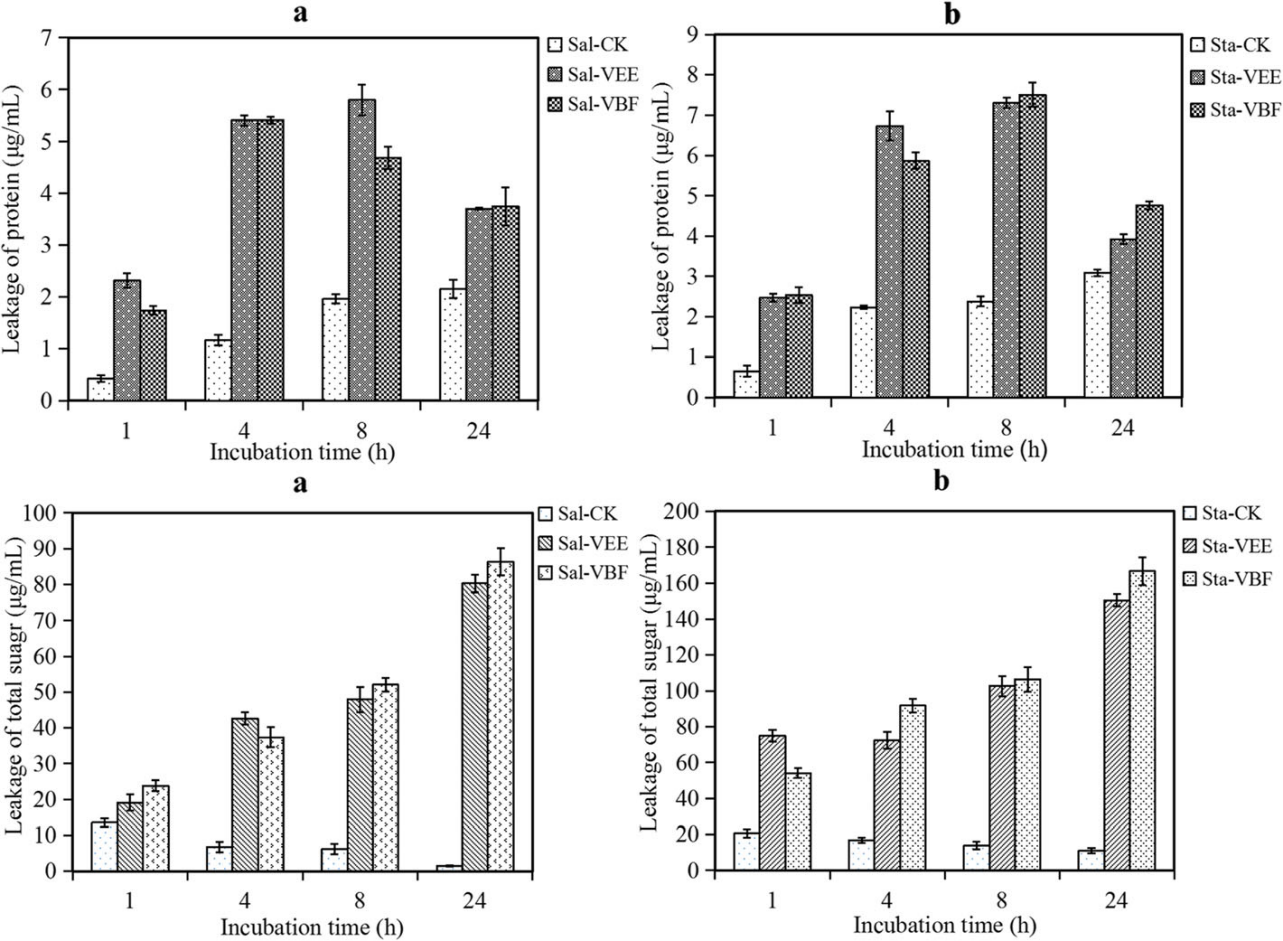
Leakage of protein and total sugar from *S. paratyphi* A (Sal, **a**) and *S. aureus* (Sta, **b**) cells treated with VEE and VBF

As shown in Fig. 2, VEE and VBF enhanced the leakage of proteins through the bacteria membrane. The leakage of proteins from *S. paratyphi* A were 2.31 μg/ml and 1.74 μg/ml when treated with VEE and VBF after 1 h incubation, which were higher than the control (0.41 μg/ml). The leakage of proteins increased with the treatment time extension. The contents of leakage proteins of *S. paratyphi* A that treated with VEE reached the highest after 8 h (5.80 μg/ml). For *S. aureus*, the leakage of proteins had the similar tendency like *S. paratyphi* A with the treatment of VEE. After 24 h incubation, the leakage of proteins decreased compared with 8 h and still higher than control. It suggested that the bacteria may entered into the decline phase and the bacteria amounts were reduced.

#### The total sugar in the medium

As Fig. 2 showed, after treated with VEE and VBF, the total sugar in the medium increased with the incubation time and reached the maximum at 24 h. The total sugar leakage of *S. paratyphi* A that treated with VEE and VBF were 80.32 and 86.32 μg/ml. For *S. aureus*, VEE caused the leakage of total sugar with the content of 150.53 μg/ml. The total sugar of *S. aureus* that treated with VBF was 166.47 μg/ml. The total sugar of the control decreased with the incubation time because the normal bacteria in growth process absorbed and utilized the sugar in the medium.

#### Leakage of nucleic acid

**Fig. 3:**
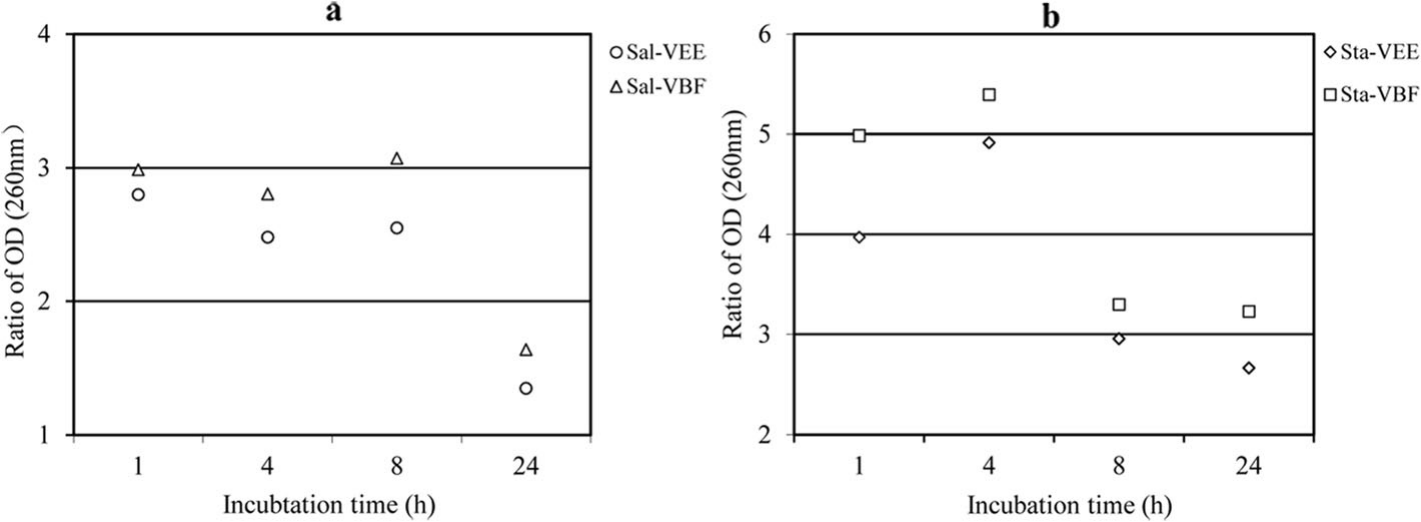
Leakage of nucleic acid from *S. paratyphi* A (Sal, **a**) and *S. aureus* (Sta, **b**) cells treated with VEE and VBF

As shown in Fig. 3. The ratio of OD that the bacteria suspension treated with VEE and VBF compared with control were plotted versus time. VEE and VBF increased the OD value to 1.64–5.39-fold compared with that of untreated bacteria. For two bacteria strains, the amount of 260 nm absorbing material released with addition of VEE was different. After 8 h incubation for *S. paratyphi* A, the amounts reached the maximum, while it was after 4 h for *S. aureus*. The amounts of 260 nm absorbing material of *S. aureus* suspension tended to decreased during incubation from 8 h to 24 h, and for *S. paratyphi* A, it was at 24 h. The decrease might be due to previously mentioned precipitation process and the adsorption of the 260 nm absorbing material on the precipitates. These precipitates were filtered out prior to the OD measurement.

### The phosphorus metabolism of bacteria

**Fig. 4:**
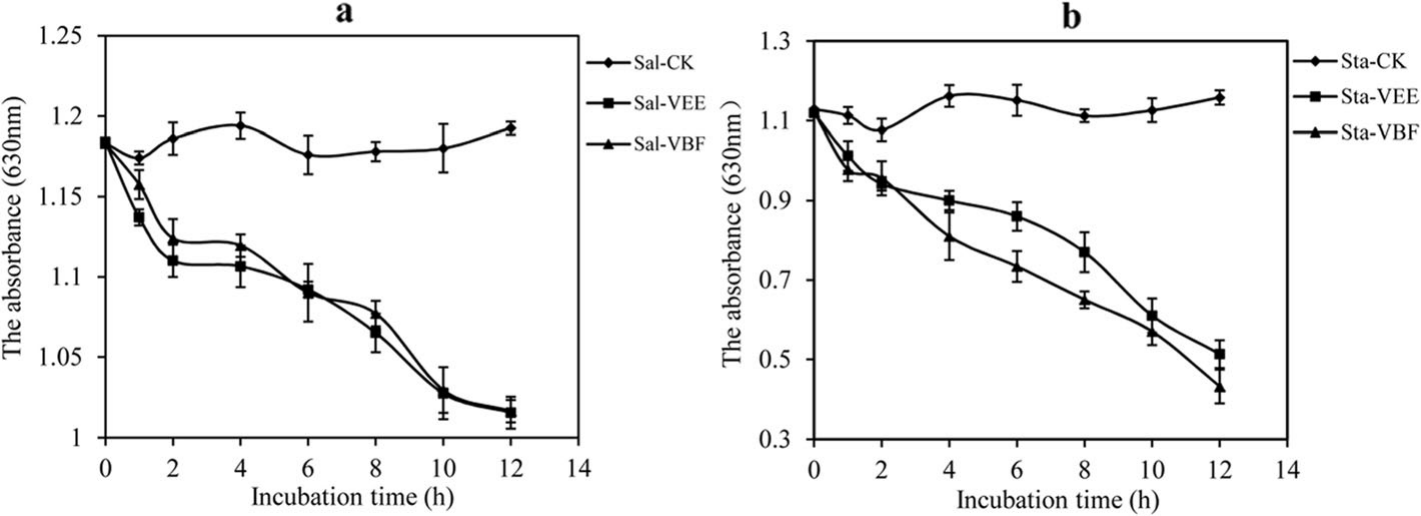
Effects of VEE and VBF on the phosphorous metabolism of *S. paratyphi* A (Sal, **a**) and *S. aureus* (Sta, **b**)

The results indicated that the consumption of phosphorus of *S. aureus and S. paratyphi* A that treated with VEE and VBF decreased with the incubation time (Fig. 4). It manifested that the phosphorus metabolism was seriously affected by the extracts and the VEE and VBF not only can destroy the membrane, but also affect the cell metabolism and growth situation.

### The protein of SDS-PAGE profiles

**Fig. 5:**
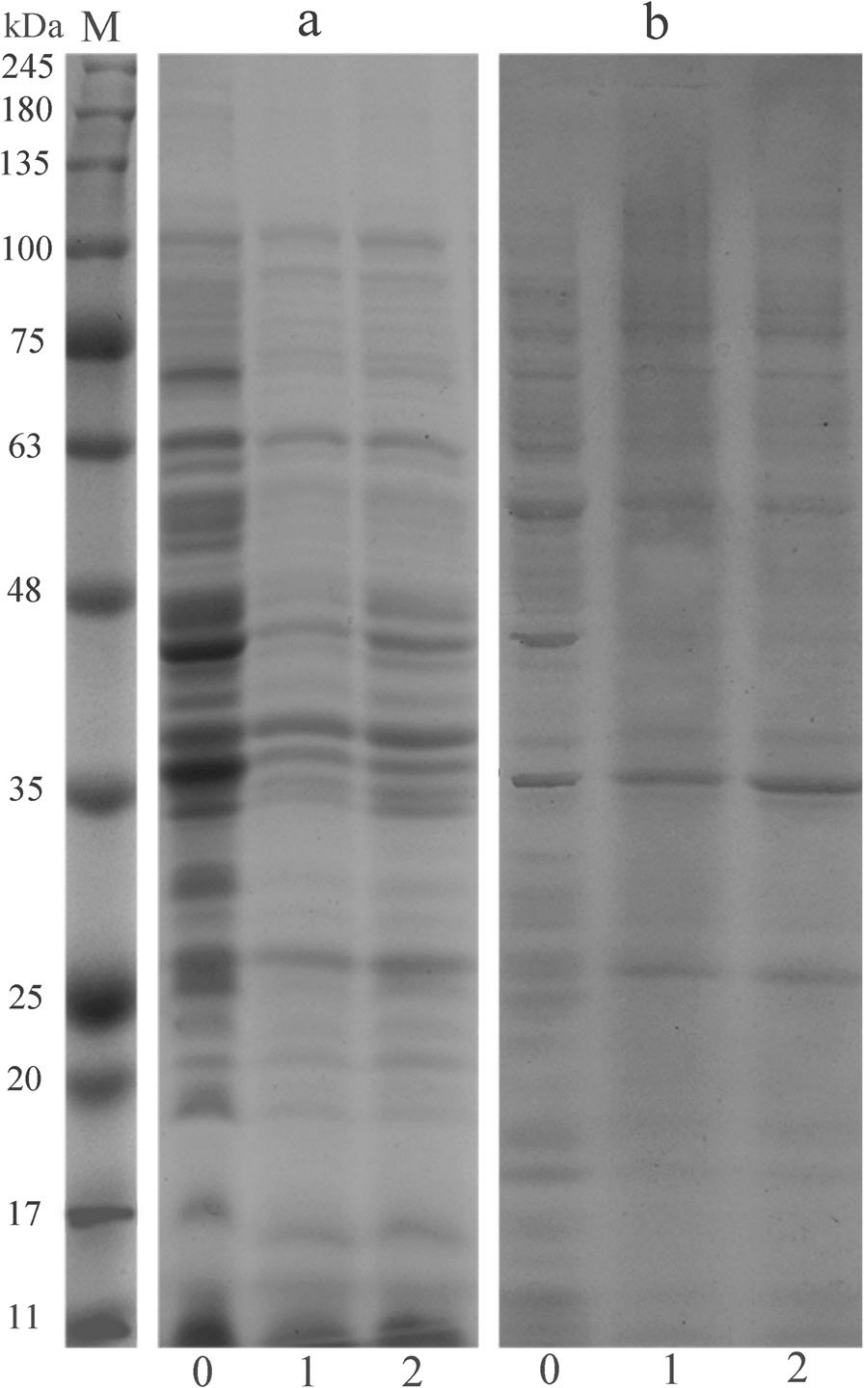
SDS-PAGE patterns of total proteins for *S. paratyphi* A and *S. aureus* treated with VEE and VBF. Note: M: marker; **a**: normal *S. paratyphi* A; **b**: normal *S. aureus*; 0: control check; 1: bacteria treated by VEE; 2: bacteria treated by VBF

As shown in Fig. 5, the protein profiles of the bacteria that treated with VEE and VBF significantly differed from that of the control. *S. aureus* and *S. paratyphi* A had more and much clearer protein bands than the control groups (a0, b0). After the treatment with the extracts at MIC value for 24 h, the bacterial protein bands faded and some even disappeared. It implied that VEE and VBF had a remarkable effect on bacterial proteins.

### Electron microscope observation

**Fig. 6:**
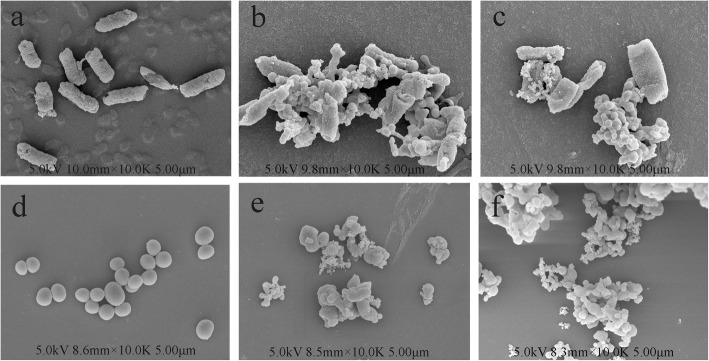
Metamorphic structure of *S. paratyphi* A and *S. aureus* treated with VEE and VBF. Note: **a**: normal *S. paratyphi* A; **b**: *S. paratyphi* A treated by VEE; **c**: *S. paratyphi* A treated by VBF; **d**: normal *S. aureus*; **e**: *S. aureus* treated by VEE; **f**: *S. aureus* treated by VBF

SEM micrographs revealed changes to the morphological and the outer surface of bacterial cells exposed to the extracts for 24 h (Fig. 6). The surfaces of all untreated cells were intact and smooth (Fig. 6a, d). *S. paratyphi* A cells untreated with the extracts had a uniform rod-shaped appearance (Fig. 6a), and *S. aureus* cells grown in the absence of the extracts had a uniform bulbous shape (Fig. 6d). In contrast, *S. paratyphi* A cells that treated with VEE and VBF showed severe morphological destruction. Some treated bacterial cells became adhesive to each other and was highly distorted (Fig. 6b, c), which may lead to the leaching out of nutrient and genetic materials. For *S. aureus* cells, pore formation and cell lysis were also apparent after the treatment. Disintegration of some cells and presence of debris in the vicinity of treated cells were also observed (Fig. 6e, f). The results indicated that VEE and VBF may have severe effects on the cytoplasmic membrane and cell wall. However, these changes in more details still need to be further observed.

## Discussion

As the *Fagaceae* family of plants is known to possess a large number of polyphenols [38], we determined the TPC of *Q. variabilis* since this plant also belongs to *Fagaceae*. The result differed from the other reports on the high chemical levels of phenolics of acorns from different *Quercus* species, namely *Quercus suber* [39]*.* Therefore, *Quercus suber* and *Q. variabilis* are the same genus, but to some extent, the same genus exists difference may be due to different species and extraction method.

Chromatograms of valonia and shell extracts from *Q. variabilis* were analyzed by HPLC method. Previous researchers revealed the valonia is rich tannins in which the ellagic acid and luteic acid has been widely used in medicines and foods [21, 22]. Similarly, the ellagic acid existed in the acorn of valonia of *Q. variabilis*. Moreover, theophyline and caffeic acid were detected in acorn of *Quercus suber* [15], and they also existed in valonia and shell of *Q. variabilis*, so it showed that the chemical composition of the same genus had the similarity. Since only four compounds have been found in the extracts by RP-HPLC method. The identification of the other compounds needed to be further studied by spectrum method.

However, the leaves of *Q. variabilis* contains rich triterpenoid compounds in which 9 compounds (lupeol, friedlin, β-sitosterol, daucosterol, taraxerol, 3-epicycleucalenol, 3-epicycloeucalenol-24-one, 3-epicycloeucalenyl-acetate and valiabilisic acid) were identified [20], yet none of them was detected in the valonia and shell of *Q. variabilis* in our study.

In general, the three parameters (DIZ, MIC and MBC) were used to determine the antibacterial activity [4, 6]. In our assay, the antibacterial activities of the ethanol extracts of valonia and shell against five bacteria were studied by DIZ to process the preliminary screening of strains. The *S. aureus* and *S. paratyphi* A were chosen for further research of the antibacterial activities and related mechanism. The antibacterial activities (MIC and MBC) of ten fractions (VEE, VPEF, VEAF, VBF, VWF, SEE, SPEF, SEAF, SBF, SWF) against *S. aureus* and *S. paratyphi* A were evaluated. In terms with the antibacterial activity (MIC and MBC), VEE, VBF and VWF showed similar activity. At the same time, we considered the DIZ. The result showed that VWF had the weaker antibacterial activity compared to VEE and VBF, so VWF was not chosen for further assays. VEE and VBF were chosen to further study the mechanism.

In this study, since the ellagic acid was reported with antioxidant, antibacterial and anti-inflammatory activities, we determined the antibacterial activity of ellagic acid [40]. The inhibition zones of ellagic acid against *S. aureus* and *S. paratyphi* A were 11.45 ± 0.25 mm and 10.24 ± 0.14 mm, below than that of VEE and VBF. Meanwhile, the RP-HPLC analysis indicated that ellagic acid was rich in VEAF, but the antibacterial effect of VEAF was lower than VEE and VBF. It suggested that ellagic acid was not the main antibacterial activities composition.

The cell membrane is an important part of bacteria. It plays an important role in maintaining normal bacterial life by balancing material and energy. It can stop the exogenous material entering and prevent the internal material leaking [41]. Sugar is the exogenous nutrients that provides primary carbon source and energy reserves material to bacteria. In normal physiological situation, the bacteria can absorb and utilize the exogenous nutrients, and the cellular content would leak after the membrane structure was damaged [33], so evaluation of cell leakage markers including absorbance at 260 nm for nucleic acid and determination of protein and sugar are indicators of membrane integrity. The leakage of nucleic acid through the bacteria membrane and the total sugar in the medium indicated that VEE and VBF had effects on the membrane of bacteria. The results of the leakage of sugar and nucleic acid were in agreement with some other antimicrobials treated on bacterial cells [33, 42].

Phosphorus is the necessary microelement and important constituent part of nucleic acid, phospholipid and sugar metabolism of microorganism and the cellular energy metabolism. The bacteria could utilize the glucose and go through a series of phosphorylation to provide the energy needed [33]. It is as expected that this is consistent with our results. The normal transportation of secretory proteins plays an important role in the growth of bacteria and in the life activity of bacterial cells [5]. When *S. aureus* and *S. paratyphi* A were treated with VEE and VBF for a certain time, protein contents in the supernatant were higher than their respective control. It stated that VEE and VBF cause the permeabilizing action. To further confirm it and to better understand the action, *S. aureus* and *S. paratyphi* A were further analyzed by SDS-PAGE for their soluble protein. The results indicated that VEE and VBF had a remarkable effect on bacterial proteins either by inhibiting their synthesis or destroying them, resulting in their death. SEM micrographs can intuitively reflect the morphological alterations of the bacteria membrane [4]. The results indicated adverse effects of VEE and VBF towards the cell of *S. aureus* and *S. paratyphi* A as compared to the control groups. Moreover, impaired membrane structure was revealed at higher extract concentrations. It may be due to lysis of membrane and transformation caused by the damage on the permeability and integrity of membrane from the samples.

## Conclusions

The results indicated that valonia and shell of *Q. variabilis* possessed higher content of phenolic compositions and tannins, in which the ellagic acid is abundant in valonia extracts. VEE and VBF showed the best antibacterial activities against *S. paratyphi* A and *S. aureus* and the results of antibacterial mechanism showed that the extracts could damage the membrane of bacteria and result in great leakage of protein and nucleic acid of bacterial cells as well as the total sugar in the medium. Measurements of the SDS-PAGE profiles confirmed the disruptive action of the on cytoplasmic membrane. Meanwhile, the damage of cell membrane was in accordance with that of SEM. It could be conferred that integrity of cell membrane would be an important factor to inhibit the bacterial normal growth and cellular metabolism. Therefore, this study suggested possible applications of valonia from *Q. variabilis* as natural antimicrobials or preservatives in the food and medicine field. In this study, ellagic acid was not the main antibacterial material so the antibacterial substances of VEE and VBF need to be further extracted, isolated and identified. Further research on molecular cytology level was needed to comprehensively investigate the antibacterial mechanism and find the specific target of substance, which could provide the theoretical basis for development and utilization of *Q. variabilis* resources.

## Data Availability

The datasets supporting the conclusions of this article are included within the article. The voucher specimen was identified by the professor Dengwu Li and was deposited at Herbarium of the Northwest A&F University, Yangling, China (WUK 0489424).

## Abbreviations

*L. monocytogenes*: *Listeria monocytogenes*
MBC: Minimum bactericidal concentration.
MIC: Minimum inhibition concentration
*Q. variabilis*: *Quercus variabilis*
*S. aureus*: *Staphylococcus aureus*
*S. enteritidis*: *Salmonella, L. enteritidis*
*S. paratyphi* A: *Salmonella, paratyphi* A
*S. typhimurium*: *Salmonella typhimurium*
SBF: the n-butanol fraction of shell
SEAF: the ethyl acetate extracts of shell
SEE: the ethanol crude extract of shell
SEM: scanning electron microscopy
SPEF: the petroleum ether extracts of shell
SWF: the water extracts of shell
TFC: the total flavonoid content
TPC: the total phenolic content
TTC: the total tannin content
VBF: the n-butanol fraction of valonia
VEAF: the ethyl acetate extracts of valonia
VEE: the ethanol crude extracts of valonia
VPEF: the petroleum ether extracts of valonia
VWF: Water fraction of valonia
